# Active Components of Fungus *Shiraia bambusiscola* Can Specifically Induce BGC823 Gastric Cancer Cell Apoptosis 

**DOI:** 10.22074/cellj.2016.4309

**Published:** 2016-05-30

**Authors:** Shubing Zhang, Dewen Qiu, Jingjiang Liu, Zhijian Li

**Affiliations:** 1 Department of Anesthesiology, The Second Xiangya Hospital of Central South University, Changsha, Hunan, China; 2 Department of Cell Biology, State Key Laboratory of Medical Genetics and School of Life Sciences, Central South University, Changsha, Hunan, China

**Keywords:** *Shiraia Bambusicola*, Apoptosis, PARP, FLIP, Gastric Cancer

## Abstract

**Objective:**

Gastric cancer is a major health issue worldwide. Using a therapeutic approach, with minor side-effects, is very essential for the treatment of the gastric cancer. *Shiraia bambusicola* is a parasitic fungus which is widely used in China for curing several diseases with little side-effects. However, the mechanisms are not well understood yet. The aim of this study was to further understand the pharmacological mechanisms of *Shiraia bambusicola* and investigate whether it can be used for curing gastric cancer.

**Materials and Methods:**

In this experimental study, we mainly tested the effect of active components extracted from *Shiraia bambusicola* on BGC823, A549 and HepG2 cells. We used MTT assay to test cell viability. We also analyzed morphologic changes caused by apoptosis using Hoechst 33342 fluorescence staining, as well as cell cycle status and apoptosis ratio using flow-cytometer. In addition, protein expression level was tested by Western-blotting assay.

**Results:**

BGC-823 cell proliferation was specifically inhibited by active components of *Shiraia bambusicola*. Meanwhile, these active components could induce BGC-823 cells apoptosis and retard the cell cycle in S/G2 phase. We also determined that two critical protein markers cleaved Poly(ADP-ribose) polymerase-1 (PARP-1) and FLICE-inhibitory protein (FLIP), involved in apoptosis process, were regulated by these active components.

**Conclusion:**

These data shed light on the treatment of human gastric cancer and conclude that *Shiraia bambusicola* can be a good therapeutic candidate for treatment of this malignancy.

## Introduction

Gastric cancer can be initiated in any part of the stomach and may spread throughout this organ and many others, particularly esophagus and the small intestine (1). This type of malignancy, as the most common cause of cancer-related death, is a major health issue worldwide. Although, this malignancy represents roughly 2% (21,500) of all new diagnosed cancer cases, every year, in the United States, it is much more common in Asia, including Japan and China (2,3). Investigations report that this disorder is associated with using high amount of salt in diet, smoking, as well as low intake of fruits and vegetables (4). 

Prognosis of the patients with gastric cancer is related to tumor extent, including both nodal involvement and direct tumor extension beyond the gastric wall. Currently different types of therapeutic approach are utilized for treatment of this disease including surgery, chemotherapy and neo-adjuvant, adjuvant, and palliative therapies. Using the choice of surgical approach in gastric cancer depends on the size, location, and locally invasive characteristics of this malignant tumor (5). Current anti-neoplastic or combinational therapeutic agents, such as Epirubicin, Cisplatin, 5-FU, Docetaxel and Irinoteca, used in managing gastric cancer have very strong side-effects on the health of patients (6). Therefore, it is very necessary to find new therapeutic agents to treat gastric cancer disease with less side-effects (7). 

*Shiraia bambusicola* P. Henn. is a parasitic fungus on twigs of several kinds of bamboos, including *Brachystachyum densiflorum* and relevant species in China as well as Bambusa species in Japan (8,9). Generally, *Shiraia bambusicola* can cause bambusicolous disease, leading to reverse effects on plant growth. It is noteworthy to indicate that sporophores of this fungus are widely used in the southern part of China for the treatment of inflammation, apoplexy and sciatica with little side-effect representations. The chemical constituents of *Shiraia bambusicola* include several bioactive compounds, such as ergosterol, hypocrellins, 1,8-dihydroxy anthraquinone and dideoxyverticillin (10). However, the function of the active components of *Shiraia bambusicola* is not well understood yet. Previous results showed that hypocrellins extracted from Shiraia bambusicola are photosensitizers, acting as anti-tumor, anti-biotic, anti-viral and anti-inflammatory properties (8). In addition, it has been demonstrated that 11,11'-dideoxyverticillin isolated from Shiraia bambusicola has anti-angiogenic activity (11). Regarding the low side-effects and considerable efficiency, it is very important to know whether these active components obtained from Shiraia bambusicola can potentially be used for treatment of cancer, due to their low side-effects. In fact, investigating the effect of these active components on malignant cells and their mechanism of actions could be a beneficial strategy to find better approach for treatment of this type of cancer (12). 

Poly(ADP-ribose) polymerase-1 (PARP-1) is a nuclear chromatin-associated enzyme involved in several important cellular processes, particularly in the DNA repair system (13). Within apoptosis, cleaved PARP-1, as a crucial protein which could be involved in DNA degradation and used as a biomarker for detection of this procedure, is up-regulated (14). Moreover, FLICE-inhibitory protein (FLIP) is a novel determined apoptosis marker, potently blocking TNFrelated apoptosis-inducing ligand (TRAIL)-mediated cell death by interfering with caspase-8 activation (15,16). Pharmacologic down-regulation of FLIP protein could serve as a therapeutic intervention to sensitize cancer cells to apoptosis induced by TRAIL (17). Previous findings showed that both PARP-1 and FLIP play very critical roles in the apoptosis process of gastric cancer cells (18,19). 

In the present research, using human gastric cancer cell line (BGC823), lung carcinoma cell line (A549) and hepatocellular carcinoma cell line (HepG2), we attempted to investigate the effects of recruited active components from Shiraia bambusicola on different cell models as well as the potential mechanisms involved in these processes. 

## Materials and Methods

### Extraction of Shiraia bambusicola

This experimental study was approved by the Ethical Committee of Second Xiangya Hospital of Central South University. The *Shiraia bambusicola* was milled and mixed with freshly prepared 80% ethanol (EtOH, Dingguo, China) in erlenmeyer flask, followed by smoothly stirring in a digital orbital shaker for 6 hours at 70˚C. After filtering the mixture and discarding debris, the solvent named group 1 was filtrated in a rotary evaporator. When incubated with cells, the resulting solid was suspended in 80% EtOH and preserved in a concentration of 5 mg/ml. Group 1 component was further extracted by Polyamide column chromatography and the solvent was mixed with 95% EtOH, alternatively named group 3. When incubated with cells, the resulting solid (group 3) was suspended in 80% EtOH and preserved in a concentration of 2 mg/ml. 

### Cell culture

BGC823 cell line was purchased from the Shanghai Cell Collection (China). In addition, A549 and HepG2 cell lines were prepared from American Type Culture Collection (ATCC, USA). Cells were cultured according to previously described method (20). The cells were cultured in Dulbecc’s modified Eagle’s medium (DMEM, Thermo Scientific, USA) supplemented with 10% fetal bovine serum (Sijiqing, China) at 37˚C with antibiotics, and were maintained in a humidified environment containing 5% CO_2_ . 

### MTT assay

MTT assay was used to assess cell viability. The cells were grown in a 96-well plate for 48 hours, with a density of 2×10^4^ cells/well. They were subsequently treated with different concentrations of components obtained from *Shiraia bambusicola*. At the end of treatment, the medium was carefully removed and 200 μl of medium containing 20 μl MTT [5 mg/ml in phosphate-buffered saline (PBS), Sigma, USA] was added to each well. The cells were then incubated for 4 hours at 37˚C, followed by removing the medium and adding dimethyl sulfoxide (100 µl) to each well. The absorbance of each well was read at 490 nm. 

### Cell apoptosis detection by Hoechst 33342 fluorescence staining

BGC823 cells were cultured, with a density of 5000 cells/well in 12-well plates with coverglasses (Fisher Scientific, USA) placed in advance. After treatment with different concentrations of the extracted components from *Shiraia bambusicola*, the cell culture medium was removed and the cells were fixed using 0.5 ml stationary liquid (Dingguo, China), for 10 minutes. By removing the stationary liquid, the cells were rinsed twice with PBS after 3 minutes incubation. The slides containing cells were then stained by adding 0.5 ml Hoechst 33342 (Sigma, USA) and incubation for 5 minutes, followed by three times rinsing with PBS before cell observations by microscope. In this experiment, morphological changes leading to condensed or fragmented nuclei were considered as apoptotic cells (21). 

### Western blot

For western blotting, the cells were lysed in RIPA buffer (Boston Bioproducts, USA) with protease inhibitor cocktail (Roche, Switzerland). Protein concentrations were determined using the BCA protein assay kit (Thermo Scientific, USA). Samples of cell lysate supernatant (50 μg protein) were resolved on sodium dodecyl sulfatepolyacrylamide gel electrophoresis (SDS-PAGE) and electro-transferred into nitrocellulose membranes. The membranes were then probed at 4˚C overnight with the following dilutions of the indin cated antibodies: cleaved-PARP (Abcam 1:1000, UK) and FLIP (Abcam 1:1000, UK). Anti-rabbit secondary antibodies (Santa Cruz Biotechnology, USA) were used at a 1:4000 dilution. Immunocomplexes were detected by incubating with enhanced chemiluminescence (ECL, Thermo Scientific, USA) and subsequent exposure to x-ray film. Anti-β-actin polyclonal antibodies (Sigma 1:4000, USA) were incubated with the same membrane, following the membrane stripping. 

### Flow-cytometer assay

The cells were harvested and prepared as single cell suspension in washing buffer. They were then washed twice and re-suspended in a 2×10^6^cells/ml concentration. 1 ml of the cells were transferred into a 15 ml polypropylene tube on ice and 3 ml cold (20˚C) absoo lute ethanol was added. The cells were incubated for at least 1 hour at 4˚C, for fixation. They were subsea quently washed twice with PBS. Later, 1 ml of propidium iodide (PI, 40 μg/ml PI in PBS) staining solution was added to the cell pellets and mixed well. Ultimately, cell cycle and apoptosis rate of the BGC823 cells was analyzed by flow-cytometer (FACSCalibur, BD, USA) using manufacturer’s protocol. 

### Statistical analysis

Statistical analysis of the results was carried out using one-way ANOVA as well as Student’s t test. P<0.05 was considered statistically significant. Data from all experiments are presented as mean ± SD of at least three independent experiments, performed in duplicate for each construct. 

## Results

### BGC-823 cell proliferation can specifically be inhibited by active components of Shiraia bambusicola

**Fig.1:**
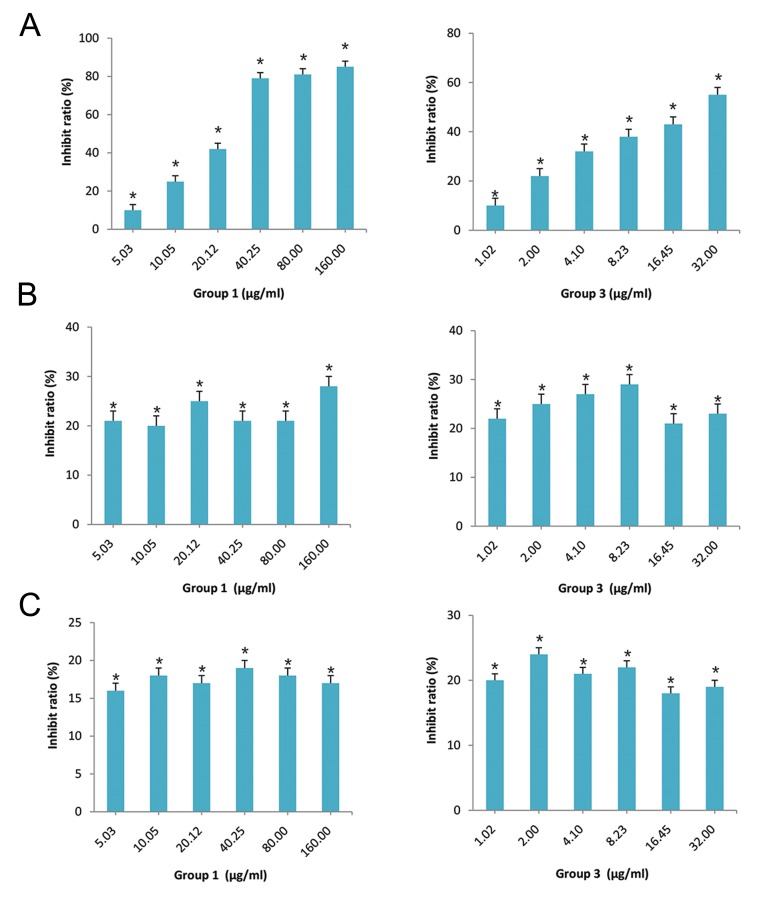
Active components of *Shiraia bambusicola* can specifically inhibit BGC823, but not A549 and HepG2 cell proliferations. Using MTT assay, the images show cell growth inhibition of A. BGC823 cells, B. A549 cells, and C. HepG2 cells, induced by group 1 and group 3 at different concentrations. *; P<0.05, n=6.

BGC823, A549 and HepG2 cell lines were considered as good models *in vitro* to investigate potential treatment strategy for human gastric cancer, lung carcinoma and hepatocellular carcinoma, respectively. Using BGC823, A549 and HepG2 cells treated with extracts of *Shiraia bambusicola*, we found that only BGC823 proliferation can be inhibited by the active components group 1 and group 3 (Fig.1A). These active components have no effect on A549 and HepG2 cells growth (Fig.1B,C). Meanwhile, we found that BGC823 cells proliferation can be inhibited by group 3 in a lower rate than group 1 (Fig.1A), confirming the fact that group 3 is the component extracted from group 1. 

### Active components of Shiraia bambusicola can induce BGC-823 morphological cell changes and apoptosis 

**Fig.2:**
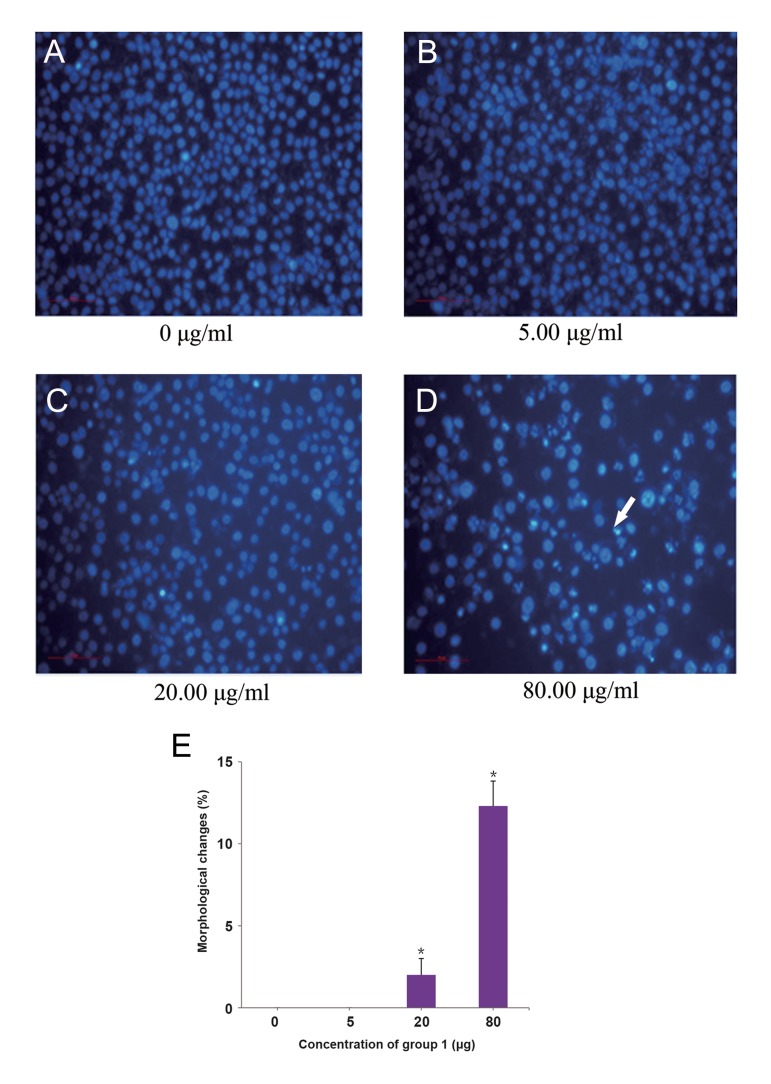
Morphological changes of BGC823 cells treated with different concentrations of group 1 obtained from *Shiraia bambusicola*, using Hoechst 33342 fluorescence staining. These morphological changes were analyzed in the treated cells with A. 0 μg, as negative control, B. 5 μg, C. 20 μg, D. 80 μg concentration of group 1, and E. Image shows percentage of the cells with morphological changes. White arrow shows the condensed nucleus cell. *; P<0.05.

**Fig.3:**
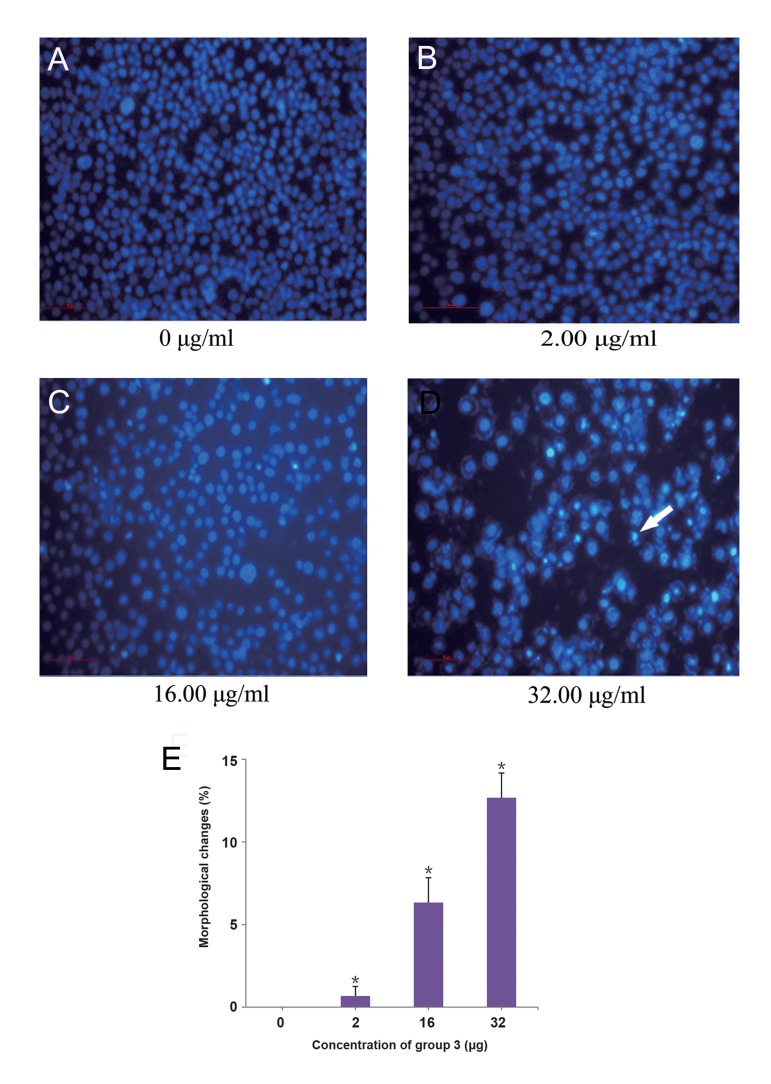
Morphological changes of BGC823 cells treated with different concentrations of group 3 obtained from *Shiraia bambusicola*, using Hoechst 33342 fluorescence staining. These morphological changes were analyzed in the treated cells with A. 0 μg, as negative control, B. 2 μg, C. 16 μg, D. 32 μg concentration of group 3, and E. Image shows percentage of the cells with morphological changes. White arrow shows the condensed nucleus cell. *; P<0.05.

Human gastric cancer is one of the most common malignant tumors with a high rate of death. Using gastric cancer cell line, BGC823, we have already determined that active components group 1 and group 3 could prevent cell growth. Subsequently, we investigated whether these active components can induce cell apoptosis in BGC823 cells, using Hoechst 33342 fluorescence staining. Findings showed a significant change in the morphology of cells treated with different concentration of active components, in a dosedependent manner (Fig.2A-E,3A-E). In this context, several visible condensed or fragmented nuclei were observed by treating the cells with 80 μg of group 1 (Fig.2D), or 32 μg of group 3 extract (Fig.3D), while the former cells (containing condensed nuclei) were determined as apoptotic cells (Fig.2D,3D,whitearrows). 

### Cell cycle was retarded in S/G2 phase and apoptosis ratio was increased by treatment of BGC-823 cells with group 3 at high concentration 

**Fig.4:**
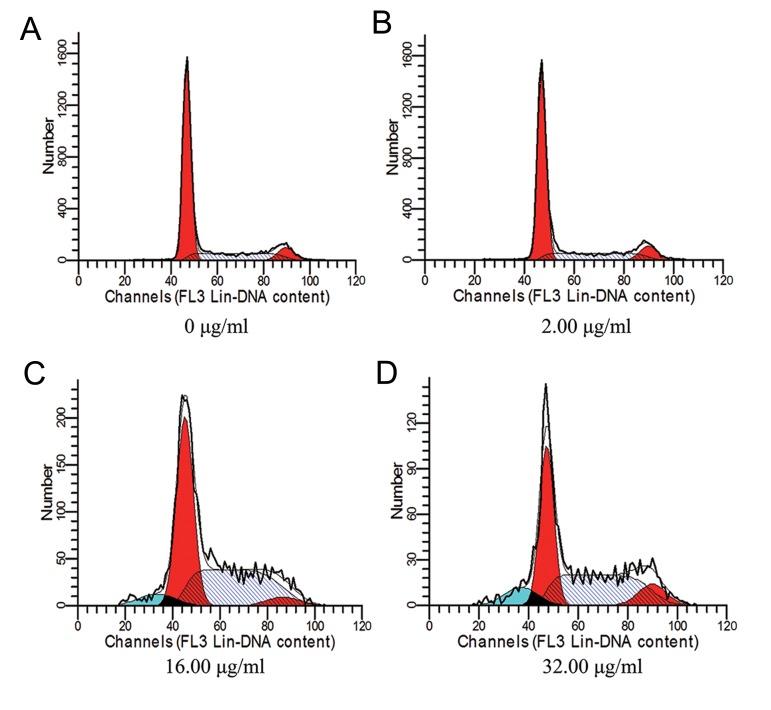
The representative image of flow-cytometer assay results. Images show the BGC-823 cells while they were treated with A. 0 μg, as negative control, B. 2 μg, C. 16 μg, and D. 32 μg of group 3 extracts.

Although both group 1 and group 3 can prohibit BGC823 cell growth and induce apoptosis, we focused on study of active components group 3 as the candidate of therapeutic approach, since it was extracted from group 1. Using flow-cytometer, we found that BGC823 cell cycle was retarded in S/G2 phase (Fig.4,5A). Meanwhile, the apoptosis ratio of BGC823 cells was also elevated, by gradually increasing concentration of group 3 extract treatment (Fig.4,5B). These results implicated that group 3 could have an effect on both cell cycle and apoptosis processes at BGC823 cells, suggesting that active components group 3 could be a good candidate for the treatment of gastric cancer. 

**Fig.5:**
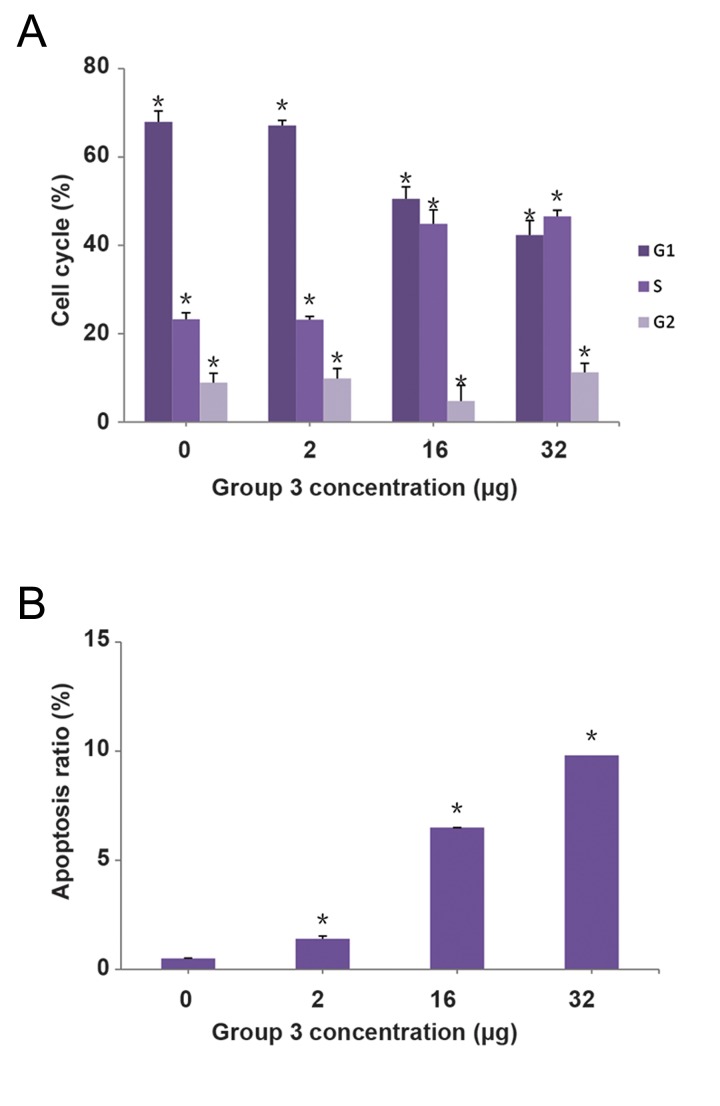
Images represent the percentage of A. Cell cycle phase blockage and B. Apoptosis ratio in the BGC-823 cells treated with 0, 2, 16 and 32 μg of the group 3 extracts.*; P<0.05

### Treatment with group 3 can affect cleaved PARP-1 and FLIP protein expression levels in BGC-823 cells

**Fig.6:**
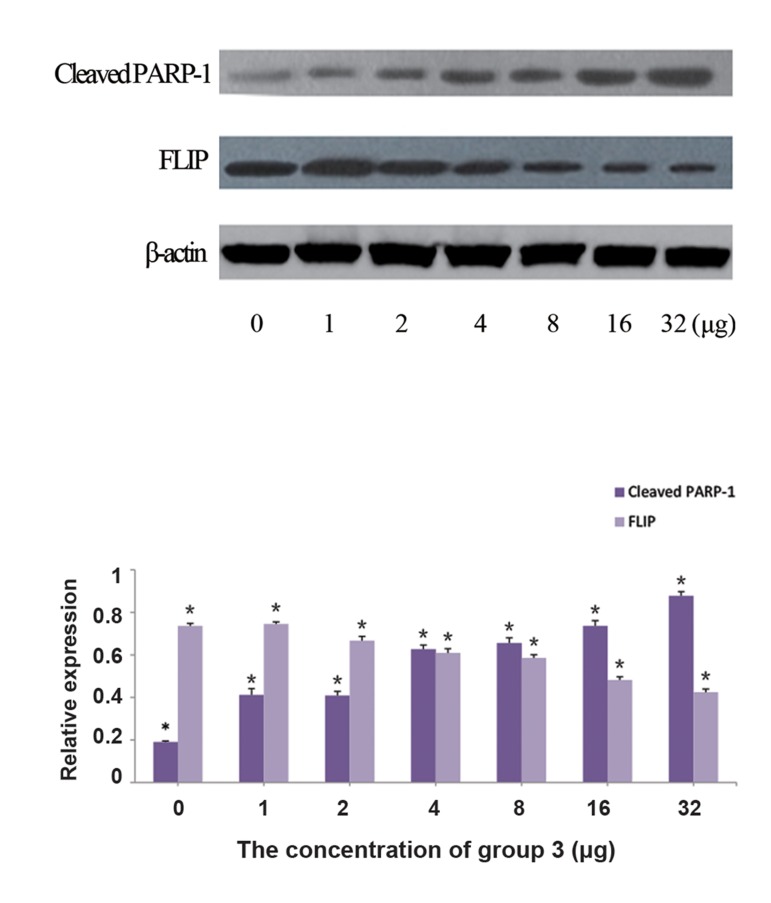
mages show the A. Qualitative and B. Quantitative effects of cleaved PARP-1 and FLIP protein expression levels in BCG823 cells treated with different concentrations (0, 1, 2, 4, 8, 16 and 32 μg) of group 3, using western blotting assay. In this experiment, two primary antibodies were employed to determine cleaved PARP-1 as well as FLIP. β-actin was also used as internal control. *; P<0.05.

To further understand the mechanism involved in the physical process of BGC823 cells induced by group 3, we tested the protein levels of cleaved PARP-1 and FLIP by western-blotting assay. Regarding that both of these proteins are crucial markers for apoptosis, the aim of this study was to find out whether cleaved PARP-1 and FLIP protein expression levels were modulated during BGC823 cell apoptosis, due to treatment with group 3. Findings demonstrated, respectively up-regulation and down-regulation of cleaved PARP-1 and FLIP protein levels in BGC823 cells treated with gradually increasing concentration of group 3, 0-32 μg (Fig. 6A, B).

## Discussion

Fungus *Shiraia bambusicola* has been used for the treatment of different diseases for long time, among Chinese population. Thus far, very rare side-effects have been determined from this parasite, turning that into a reliable treatment approach for long time administration. Previous reports implicated on several active components isolated from sporophores of *Shiraia bambusicola* (8), however, the functions of these active components are not clear yet. Regarding that many classical chemotherapy agents cause strong side-effects on patients, it is crucial to find novel agents with minor side-effects for the treatment of gastric cancer. 

In this experiment, we used different types of human cancer cell line, as *in vitro* models, to investigate the function of active components isolated from *Shiraia bambusicola*. Our results showed that both group 1 and group 3 can specifically inhibit proliferation and stimulate apoptosis of the BGC823 cells. These results suggest that *Shiraia bambusicola* could be a candidate for human gastric cancer treatment. Since group 3 is a component extracted from group 1, the percentage of useful materials from group 3 may be higher compared to group 1. Hence, we focused on the effects of group 3 on BGC823 cell cycle blockage and apoptosis. Our findings confirmed that group 3 can dose-dependently promote BGC823 cell apoptosis and retard cell cycle in the S/G2 phase. 

Cell apoptosis is a process of the programmed cell death that may occur in multicellular organisms and be involved in many biochemical events, leading to characteristic cell morphology changes and death. Many critical genes, such as P53, Bcl_2_, Caspases, PARP-1 and FLIP can participate in apoptosis process (22,23). Therefore, we focused on two apoptotic markers, PARP-1 and FLIP, to determine their effects in apoptosis of the treated BGC823 cells with group 3. Our findings showed that cleaved PARP-1 protein level was increased, while the expression of FLIP protein was decreased. These data confirmed that both PARP-1 and FLIP are key proteins, during the apoptosis process of BGC823 cells induced by the group 3. 

It is very promising that the active components of *Shiraia bambusicola* can induce BGC823 apoptosis and we propose that *Shiraia bambusicola* might be a good candidate for treatment of human gastric cancer. However, it is noteworthy to indicate that these active components could only have effect on some types of gastric cancer. Further investigations are required *in vitro* and *in vivo* to determine the precise effect of *Shiraia bambusicola* on human gastric cancer. 

## Conclusion

Together, our data shed light on the treatment of human gastric cancer. The active components of *Shiraia bambusicola*, which can inhibit BCG823 proliferation, block cell cycle on S/G2 phase and induce cell apoptosis, might be useful for treatment of this malignancy. Meanwhile, we confirmed that PARP-1 and FLIP proteins play important roles during apoptotic process of the BGC823 cells, induced by active components of *Shiraia bambusicola*. 

## References

[1] Hu Y, Wang J, Qian J, Kong X, Tang J, Wang Y (2014). Long noncoding RNA GAPLINC regulates CD44-dependent cell invasiveness and associates with poor prognosis of gastric cancer. Cancer Res.

[2] Choi YY, An JY, Kim HI, Cheong JH, Hyung WJ, Noh SH (2014). Current practice of gastric cancer treatment. Chin Med J (Engl).

[3] Liu L, Wang S, Cao X, Liu J (2014). Diagnostic value of circulating microRNAs for gastric cancer in Asian populations: a meta-analysis. Tumour Biol.

[4] Song P, Wu L, Guan W (2015). Dietary nitrates, nitrites, and nitrosamines intake and the risk of gastric cancer: a metaanalysis. Nutrients.

[5] De Silva N, Schulz L, Paterson A, Qain W, Secrier M, Godfrey E (2015). Molecular effects of Lapatinib in the treatment of HER2 overexpressing oesophago-gastric adenocarcinoma. Br J Cancer.

[6] Onoyama M, Kitadai Y, Tanaka Y, Yuge R, Shinagawa K, Tanaka S (2013). Combining molecular targeted drugs to inhibit both cancer cells and activated stromal cells in gastric cancer. Neoplasia.

[7] Li Y, Wang K, Jiang YZ, Chang XW, Dai CF, Zheng J (2014). 2,3,7,8-Tetrachlorodibenzo-p-dioxin (TCDD) inhibits human ovarian cancer cell proliferation. Cell Oncol (Dordr).

[8] Fang LZ, Qing C, Shao HJ, Yang YD, Dong ZJ, Wang F, et al (2006). Hypocrellin D, a cytotoxic fungal pigment from fruiting bodies of the ascomycete Shiraia bambusicola. J Antibiot (Tokyo).

[9] Du W, Liang J, Han Y, Yu J, Liang Z (2015). Nitric oxide mediates hypocrellin accumulation induced by fungal elicitor in submerged cultures of Shiraia bambusicola. Biotechnol Lett.

[10] Tong Y, Zhang X, Zhao W, Zhang Y, Lang J, Shi Y (2004). Anti-angiogenic effects of Shiraiachrome A, a compound isolated from a Chinese folk medicine used to treat rheumatoid arthritis. Eur J Pharmacol.

[11] Chen Y, Zhang YX, Li MH, Zhao WM, Shi YH, Miao ZH (2005). Antiangiogenic activity of 11,11'-dideoxyverticillin, a natural product isolated from the fungus Shiraia bambusicola. Biochem Biophys Res Commun.

[12] Li W, Sharma M, Kaur P (2014). The DrrAB efflux system of Streptomyces peucetius is a multidrug transporter of broad substrate specificity. J Biol Chem.

[13] Eustermann S, Wu WF, Langelier MF, Yang JC, Easton LE, Riccio AA (2015). Structural basis of detection and signaling of DNA single-strand breaks by human PARP-1. Mol Cell.

[14] Kashima L, Idogawa M, Mita H, Shitashige M, Yamada T, Ogi K (2012). CHFR protein regulates mitotic checkpoint by targeting PARP-1 protein for ubiquitination and degradation. J Biol Chem.

[15] Roth W, Reed JC (2004). FLIP protein and TRAIL-induced apoptosis. Vitam Horm.

[16] Wang W, Zhao J, Wang H, Sun Y, Peng Z, Zhou G (2010). Programmed cell death 4 (PDCD4) mediates the sensitivity of gastric cancer cells to TRAIL-induced apoptosis by down-regulation of FLIP expression. Exp Cell Res.

[17] Zhang DW, Li HY, Lau WY, Cao LQ, Li Y, Jiang XF, et al (2014). Gli2 silencing enhances TRAIL-induced apoptosis and reduces tumor growth in human hepatoma cells in vivo. Cancer Biol Ther.

[18] Zhang YS, Xie JZ, Zhong JL, Li YY, Wang RQ, Qin YZ (2013). Acetyl-11-keto-β-boswellic acid (AKBA) inhibits human gastric carcinoma growth through modulation of the Wnt/β-catenin signaling pathway. Biochim Biophys Acta.

[19] Belkhiri A, Zhu S, Chen Z, Soutto M, El-Rifai W (2012). Resistance to TRAIL is mediated by DARPP-32 in gastric cancer. Clin Cancer Res.

[20] Zhang S, Chung WC, Wu G, Egan SE, Xu K (2014). Tumor-suppressive activity of lunatic fringe in prostate through differential modulation of notch receptor activation. Neoplasia.

[21] Wang G, Feng CC, Chu SJ, Zhang R, Lu YM, Zhu JS, et al (2015). Toosendanin inhibits growth and induces apoptosis in colorectal cancer cells through suppression of AKT/GSK3β/β-catenin pathway. Int J Oncol.

[22] Wang K, Li Y, Jiang YZ, Dai CF, Patankar MS, Song JS (2013). An endogenous aryl hydrocarbon receptor ligand inhibits proliferation and migration of human ovarian cancer cells. Cancer Lett.

[23] Mohan S, Abdul AB, Abdelwahab SI, Al-Zubairi AS, Sukari MA, Abdullah R (2010). Typhonium flagelliforme induces apoptosis in CEMss cells via activation of caspase-9, PARP cleavage and cytochrome c release: its activation coupled with G0/G1 phase cell cycle arrest. J Ethnopharmacol.

